# The Roles of Primary Emotional Systems and Need Satisfaction in Problematic Internet and Smartphone Use: A Network Perspective

**DOI:** 10.3389/fpsyg.2021.709805

**Published:** 2021-08-31

**Authors:** Dmitri Rozgonjuk, Kenneth L. Davis, Christian Montag

**Affiliations:** ^1^Department of Molecular Psychology, University of Ulm, Ulm, Germany; ^2^Institute of Mathematics and Statistics, University of Tartu, Tartu, Estonia; ^3^Pegasus International Inc., Greensboro, NC, United States

**Keywords:** need satisfaction, problematic smartphone use, affective neuroscience personality scales, problematic Internet use, smartphone addiction, Internet addiction, smartphone use disorder, Internet use disorder

## Abstract

Problematic Internet and smartphone use (PIU and PSU, respectively) have received significant attention over the past years. In the current work, we studied the associations between PIU and PSU, primary emotional systems, and need satisfaction. The effective sample comprised 399 people who responded to scales measuring these variables. Bivariate correlation analysis showed that both PSU and PIU were positively associated with negative primary emotion traits (FEAR, ANGER, SADNESS) as well as lower scores on most of the need satisfaction factors. Network analysis showed that while PIU and PSU have a strong association with each other, in general, there were not many significant correlations between PSU, PIU, and other variables in the network. The associations being present were rather weak. Network analysis showed that PSU was positively associated with FEAR, ANGER, PLAY primary emotional systems. Both PSU and PIU had a negative association with safety and security and physiological needs satisfaction. Moreover, PSU had a positive link with belongingness need satisfaction, while higher PIU was associated with lower esteem and self-actualization need satisfaction. Addressing those unmet needs may be helpful in reducing problematic technology use, but further research testing this would be necessary.

## Introduction

Over more than a decade, researchers have aimed to understand the interplay between smartphone use and its associations and potential effects on human behavior and psychology. While Internet and smartphone use can enhance daily-life activities and productivity, they can also have detrimental effects when used excessively. Furthermore, recent studies have demonstrated that there are individual differences in predisposing factors, such as personality traits (Carvalho et al., 2018; Marengo et al., 2020), as well as factors that overlap between different psychopathologies (also called transdiagnostic factors, such as emotion regulation, distress tolerance, etc.) that could potentially explain the engagement in excessive Internet and smartphone use (Elhai et al., 2019). The aim of this study is to investigate the interplay between primary emotional systems, need satisfaction, and problematic Internet and smartphone use.

### Problematic Internet and Smartphone Use

The beginning of problematic Internet use (PIU) research dates back to when the Internet became more widely diffused in everyday use. It has been labeled as Internet “addiction” (Young, 1998), but researchers have moved away from this terminology, treating “addiction” as a misnomer (Starcevic, 2013) and using the term “problematic Internet use” instead (Alt and Boniel-Nissim, 2018). An alternative term is Internet use disorder (Montag et al., 2021b), which is in line with the nomenclature proposed by the World Health Organization in the context of gaming disorder. In essence, this phenomenon encompasses daily-life adversities due to excessive Internet use. Similarly, with the widespread use of smartphones, associations between problems in everyday life functioning and smartphone use have been noted. As with the Internet use, researchers coined the term “problematic smartphone use” (PSU) to mark the negative associations with excessive smartphone use (Elhai et al., 2017). Although Internet and smartphone use could be in some contexts seen as synonymous, they may fuel different problematic behaviors; in fact, PIU is considered an overarching phenomenon that mediates specific online behaviors, as well as PSU (Baggio et al., 2018). Montag et al. (2021b) have also hypothesized that PSU could be viewed as a mobile form of PIU.

Both PIU and PSU have been associated with several affect-related psychopathology as well as transdiagnostic constructs. For instance, the (small-to-moderate) correlations between excessive digital technology use and depression and anxiety symptom severity are well-documented (Elhai et al., 2017, 2020a; Rozgonjuk et al., 2018). In addition, correlations have been found with variables that are closely associated with depression and anxiety, such as emotion dysregulation (Hoffner and Lee, 2015; Spada and Marino, 2017; Rozgonjuk and Elhai, 2020), fear of missing out (Hamutoglu et al., 2020; Rozgonjuk et al., 2020c; Servidio, 2021), and negative affectivity (Elhai et al., 2018; Li et al., 2020; Zeng et al., 2021). These findings may hint that individual differences in personality traits—relatively stable characteristics over time—could drive these relationships (Marengo et al., 2020).

A relevant theoretical framework that has been used to conceptualize the findings in recent works (Elhai et al., 2020b; Hussain et al., 2020; Rozgonjuk et al., 2020a) is the Interaction of Person-Affect-Cognition-Execution model of addictive behaviors (I-PACE; Brand et al., 2016, 2019). According to the I-PACE, predisposing factors (e.g., personality traits, psychopathology, genetics, etc.) interact with certain aspects of specific situations which result in experiences of gratification and compensation that are linked to a specific behavior associated with digital technology use. Over time, this behavior may be reinforced, and in some cases it could become problematic. In turn, problematic engagement in digital technology use could influence predisposing factors (e.g., elevate or trigger psychopathology, change personality traits to some extent, etc.). Here, transdiagnostic factors such as emotion dysregulation, distress tolerance, and fear of missing out could serve as mediators and moderators in association between predisposing personal characteristics and digital technology use. It is important, however, to note that while excessive digital technology use does not become problematic in all people, it is viewed as dependent on affective variables and needs satisfaction (Brand et al., 2019).

It should also be noted that the discussion on whether PIU and PSU constitute behavioral addictions has been ongoing virtually since the introduction of the Internet and smartphones to the masses (see the following works: Billieux et al., 2015; Panova and Carbonell, 2018; Montag et al., 2021b). Additionally, whether there is a claimed negative link between digital technology use and psychological well-being has been questioned recently (Orben and Przybylski, 2019); yet, there is a difference between studies focusing on *general* vs. *problematic* digital technology usage patterns (either studying the time spent online vs. symptoms related to negative affect due to one's own digital technology use). By definition, the latter is focused on daily life disturbances associated with *excessive* digital technology use. That is, PIU and PSU research focuses on one tail of usage distribution and daily-life adversities associated with it. Furthermore, as also posited in the I-PACE model (Brand et al., 2019), excessive usage may result in problematic usage patterns in only some people. We wanted to outline these nuances for the readers' guidance: it is quite common in the field of digital technology research that the results of general usage patterns are carried over to problematic usage research, although that kind of interpretation may not be justified.

### Affective Neuroscience Theory

One relatively new approach to understand why humans differ in personality stems from neuroscientific work. Based on animal research with strong causal evidence, the affective neuroscience theory posits that there are seven primary emotional systems that are anchored in phylogenetically old subcortical brain areas (Panksepp, 1991, 1998). These primary emotional systems are evolutionary tools for survival shedding light on our mammalian heritage, because these primary emotional systems are homologously conserved across the mammalian brain (Panksepp and Biven, 2012).

Panksepp (2011) carved out seven primary emotional systems with four of them linked to positive and three to negative affect. Please note, that in the following and throughout this work, primary emotional systems are written in upper case large letters to not confuse them with similar terms found in common usage or in the psychological literature. The SEEKING system energizes mammals and provides them with energy to search for a partner or food (but it also arouses other emotional systems). The LUST system is of obvious relevance for reproductive purposes and secures the continuity of a species. The CARE system triggers unconditional parenting behavior and helps to rear the offspring toward healthy adults. Finally, the PLAY system is of relevance to develop motoric skills and social competencies, initially via rough and tumble play. On the negative valence of affect, activity of the FEAR system brings a mammal out of the danger zone via flight or freezing behavior. RAGE/ANGER activates fighting in situations of frustrations, territorial conflict, but also while defending one's own offspring. The SADNESS system is triggered by separation distress, such as when a romantic couple breaks up (evolutionarily speaking, mammals are stronger in groups than alone). The primary emotional systems can be triggered by evolutionary significant events in an unconditional way, but they can also be triggered by conditioned stimuli. In this realm, it is interesting that although the mammalian brain is very complex, the activity of the positive and negative primary emotional systems can result in surprisingly simple learning patterns. Action patterns resulting in positive affect are sustained and behaviors activating negative affect are reduced.

Although all mammals have these primary emotional systems built in their brains, it has been put forward that individual differences in these systems can be observed in terms of the brain structure and functionality underlying these basic emotions (Montag and Panksepp, 2017, 2020). Such individual differences can be assessed in humans either via neuroscientific methods (e.g., magnetic resonance imaging; see Deris et al., 2017) or via a more indirect tertiary cognitive approach (via self-report): Davis et al. (2003) constructed the Affective Neuroscience Personality Scales against the background of Pankseppian Affective Neuroscience theory and linked individual differences in these primary emotional systems to the Big Five personality traits (Goldberg, 1992). For a comprehensive overview on ANPS research see Montag et al. (2021a) and a meta-analysis on links between primary emotional systems and the Big Five personality traits by Marengo et al. (2021).

With respect to the current study, the aforementioned primary emotional systems have been linked to problematic Internet and smartphone use (Montag et al., 2016) in partial correlation analysis corrected for age effects. The analyses showed that higher levels of problematic Internet use were (weakly) negatively associated with SEEKING, CARE, and PLAY, and positively associated with FEAR, ANGER, and SADNESS. For PSU, a negative association with SEEKING and positive correlations with FEAR, ANGER and SADNESS were observed. These results are in line with general findings in PSU/PIU research where problematic digital technology use has been associated with depression and anxiety (Elhai et al., 2017, 2020a)—conditions that can be characterized by negative emotional valence as well as socially passive or avoidant behavior (De Silva et al., 2005). However, the links between primary emotional systems and PSU/PIU have been observed in isolation; that is, these associations have not been controlled for the potential influence of need satisfaction.

### Satisfaction of Needs

One of the most widely discussed theories of need satisfaction was proposed by Maslow (1943, 1981). Accordingly, in his original version of the theory, Maslow (1943, 1981) proposed that basic needs drive human behavior. These basic needs are: physiological, safety and security, belongingness, self-esteem, and self-actualization.

Physiological needs refer to basic human survival related factors, such as having sufficient access to food, air, water, sleep, shelter, but also a fulfilled sex life. Need for safety and security reflects self-evidently the need to feel safe and secure in different domains (e.g., emotional, financial, etc.). Need for belongingness manifests in the need to have company, friendship, as well as intimacy. Self-esteem need reflects the desire to be respected by others and oneself. Finally, self-actualization needs encompass the realization of one's full potential (e.g., as a partner, parent, or in other endeavors).

While Maslow (1943, 1981) originally suggested that the satisfaction of those needs is hierarchical (e.g., humans must first satisfy their physiological needs, then safety and security needs, and so forth), the notion of the *hierarchical* nature of need satisfaction has not found strong empirical support (Wahba and Bridwell, 1976; Montag et al., 2020). Yet, the universality of these human needs has found validation in research (Tay and Diener, 2011). Furthermore, although Maslow's hypothetical constructs (especially their hierarchical nature) remain unvalidated, they have been operationalized by Lester (1990).

Recently it has been demonstrated that the satisfaction of Maslow's needs and the affective neuroscience theory framework can be brought together (Montag et al., 2020). Specifically, lower needs satisfaction in all domains was associated with more FEAR, ANGER, and SADNESS tendencies. In addition, higher satisfaction of all need domains was positively correlated with SEEKING and PLAY. The primary emotion of CARE was significantly positively associated with higher levels of need satisfaction in belonging, self-esteem, and self-actualization.

However, little research has been done with respect to need satisfaction and problematic smartphone/Internet use. It has previously been suggested that PSU/PIU may be associated with unmet social needs, as links between more loneliness and higher scores on PSU/PIU measures have been found (Bian and Leung, 2014; Enez Darcin et al., 2016; Costa et al., 2019). In addition, lower self-esteem has been associated with more PSU/PIU (Mamun et al., 2020; Mathew and Krishnan, 2020; Servidio, 2021; Wang and Lei, 2021). This said, the direction of causality in these associations may be more complex: it could be that unmet needs (e.g., social, self-esteem) cause higher engagement in problematic digital technology use which, in turn, could affect other needs (e.g., physiological). Pinpointing the unmet needs that correlate with problematic digital technology use may be helpful in intervention and prevention of these problematic behaviors.

### Aims of the Study

The aim of the current study is to investigate how PIU and PSU are associated with primary emotional systems and different levels of need satisfaction. Even though there is some evidence shedding light onto the associations between some of the variables, there are currently no studies that have investigated these variables in a joint framework. Yet, it is important to investigate this. On the one hand, PIU and PSU have been linked to various factors associated with negative affectivity (Evren et al., 2019; Wolniewicz et al., 2019). On the other hand, it has been hypothesized that people may engage in problematic digital technology use for mood regulation and social connectedness (Bian and Leung, 2014; Kardefelt-Winther, 2014; Brand et al., 2019). Therefore, our approach to meet the goal of this study is to provide evidence on the associations on a bivariate correlational level as well as in a multivariate, network analytic approach.

Over the past years, network analytic approaches have been increasingly applied to tackle the complexities in the interplay between psychological constructs (Borsboom and Cramer, 2013; Robinaugh et al., 2020). Network models allow us to conduct exploratory analyses, detect the structure of given psychological phenomena, visualize those complex links, and help with hypothesis generation. A network depiction, or graph, is typically a set of nodes (representing variables) and edges (depicting associations between two given nodes), with the latter indicating the association strength and also the positive or negative correlational direction of the relationship (Borsboom and Cramer, 2013; Costantini et al., 2015). Typically, partial correlations in combination with regularization techniques are used for graph generation with a set of nodes and edges (Epskamp and Fried, 2018). It is also possible to estimate each node from other nodes, resulting in the predictability of a given node expressed as *R*^2^-statistic (Haslbeck and Waldorp, 2018). Finally, the stability of network structure as well as the importance of each node in the network could be estimated. When it comes to the importance of nodes, centrality statistics can be computed. In the current work, we focus on the node strength which is the sum of all absolute edge weights of edges connected to a given node (Opsahl et al., 2010).

In the current work we aim to use this approach to (a) depict and examine the associations between PIU and PSU, primary emotional systems, and need satisfaction, (b) investigate the importance of PIU and PSU in these associations by taking a look at centrality statistics of PIU and PSU in the network model, and (c) investigate how much of PIU's and PSU's variance could be explained by the variables in the network. While there is some basis for hypothesis formulation and testing, this work is largely exploratory and could be useful in hypothesis development in subsequent studies. This said, earlier works do suggest that one may expect to find that negative primary emotional systems (FEAR, ANGER, and SADNESS) may be linked to both PIU and PSU (Montag et al., 2016). In addition, there is some evidence for expecting the links between unmet social and self-esteem needs and higher PIU and PSU.

The primary rationale for this work stems in shedding light on the associations of these variables in both bi- and multivariate manner. While some previous studies have investigated these links in isolation (e.g., PSU-PIU; primary emotional systems and PSU and PIU; need satisfaction and primary emotional systems), the current work includes all of these constructs in a single framework with the aim to account for the potential interplay between these variables. This approach is also in line with the I-PACE model (Brand et al., 2019) where primary emotional systems could be viewed as predisposing factors that may affect need satisfaction (e.g., people higher in SEEKING may have higher tendency to be social, and, hence, have their social needs satisfied, etc.). To cope with unmet needs, a person may engage in excessive digital technology use which may develop into patterns of problematic behavior. In turn, this could change the way how a person interacts with the world—and could potentially influence the person over time. Therefore, the current work could show which needs are associated with problematic technology use, as well as if some primary emotional system traits may have an interplay in that system.

## Methods

### Sample and Procedure

The sample was recruited via various media channels that included print and social media as well as the radio as a part of a larger research project. People were invited to take part in an online survey, hosted on the SurveyCoder platform (Kannen, 2018). The study was in English language and it consisted of questionnaires assessing different psychological constructs (e.g., personality, motivation) as well as digital technology use (e.g., problematic smartphone use). The current study was a part of a larger project investigating the interplay between psychological constructs and digital media use (but none of the present data have been published before). The study was voluntary and anonymous, and no financial incentive was advertised nor provided. As an incentive participants got visual feedback on their tendencies toward “smartphone addiction.” The participation in the study (e.g., filling out the questionnaires) was advertised to take ~30 min. The study was in accordance with contemporary ethical standards and it was approved by the ethics committee of the Ulm University.

In total, participation in the study was initiated in 424 cases. However, *n* = 13 cases had missing data on all variables (most likely an indication that a person clicked on the survey link, but decided not to proceed with the study after reading the information sheet). Additionally, one person did not agree to take part in the study, and since we were interested in smartphone users, we included participants who reported having a smartphone (remaining *n* = 402). We also excluded one participant who marked their age to be 441 which is implausible. Finally, we removed the participants who responded to all 24 items of ANPS-AR with the same response option consecutively, indicating careless response patterns (Curran, 2016).

The result of this data cleaning procedure was the effective sample of *n* = 399 participants [age M = 26.29, SD = 7.59, range: 18–55; 295 (73.93%) men, 104 (26.07%) women]. With regards to education level, 12 (3.01%) participants reported having less than a high school degree, 122 (30.58%) reported having a high school degree, 69 (17.29%) participants had some college education (but not a degree), 108 (27.07%) had a Bachelor's degree, 82 (20.55%) had a Master's degree, and six (1.50%) people reported having a doctoral degree. Since this study was not bound to participants from a specific country, and it was possible to take part in the study if the participant had sufficient proficiency in English language, the demographic composition of the sample included several countries. The largest number of participants was from France (88 participants, 22.06% of the total sample). Poland (30/7.52%), Austria (28/7.02%), Spain (27/6.77%), Germany (24/6.02%), Argentina (21/5.26%), and Russia (20/5.01%). Several more countries were represented in the sample, but since all other countries were reported by less than 20 people, these countries are not listed separately here.

### Measures

In addition to socio-demographic variables, such as age, gender, and education level, the participants responded to scales assessing problematic smartphone and Internet use, primary emotional systems, and basic need satisfaction. Below, we describe these inventories alongside their psychometric properties. In addition to reporting internal consistency statistics (Cronbach's alphas and McDonald's total omegas), we also present confirmatory factor analysis results indicating the goodness of the fit of models the scales aim to reflect. The procedure for the latter analyses are described in the Analysis section.

#### Problematic Smartphone Use

The short Smartphone Addiction Scale (SAS-SV; Kwon et al., 2013) was used to assess the symptom severity of PSU. SAS-SV reflects the severity of daily life adversities due to excessive smartphone use (e.g., problems at work and with concentration, symptoms of physical health issues). The responses for the ten-item SAS-SV range from 1 = strongly disagree to 6 = strongly agree. The scores of SAS-SV are summed. The internal consistency for the effective sample is Cronbach's α = 0.91 and McDonald's omega total ω_t_ = 0.91. The CFA for the scale showed an acceptable fit, χ2_(35,N=399)_ = 119.655, *p* < 0.001, CFI = 0.994, TLI = 0.992, RMSEA = 0.078 (90% CI: 0.063–0.093).

#### Problematic Internet Use

The short, 12-item version of Young's Internet Addiction Test (Pawlikowski et al., 2013) was used to assess the levels of PIU. In the present study, this instrument uses a six-point scale, with responses ranging from 1 = never to 6 = always. The item responses were summed to form a PIU score. Cronbach's α for the effective sample was 0.88 and McDonald's omega total ω_t_ = 0.88. The CFA for the scale showed an acceptable fit, χ2_(54,N=399)_ = 185.763, *p* < 0.001, CFI = 0.984, TLI = 0.981, RMSEA = 0.078 (90% CI: 0.066–0.091).

#### Primary Emotional Systems

To assess the primary emotion systems we used the 24-item Affective Neuroscience Personality Scales (ANPS-AR; Montag and Davis, 2018). ANPS-AR assesses the extent of six primary emotional traits (SEEKING, FEAR, CARE, ANGER, PLAY, SADNESS) on a seven-point response scale (1 = very inaccurate to 7 = very accurate). Cronbach's α-s for the effective sample were: α = 0.56/ω_t_ = 0.56 (SEEKING), α = 0.85/ω_t_ = 0.86 (FEAR), α = 0.69/ω_t_ = 0.70 (CARE), α = 0.55/ω_t_ = 0.59 (ANGER), α = 0.73/ω_t_ = 0.76 (PLAY), and α = 0.75/ω_t_ = 0.76 (SADNESS). The CFA showed that the six-factor solution had an acceptable fit, χ2_(237,N=399)_ = 1,356.309, *p* < 0.001, CFI = 0.930, TLI = 0.918, RMSEA = 0.109 (90% CI: 0.103–0.115).

We would like to point out that some of the ANPS-AR scales exhibit poor internal consistency. This may also be due to the fact that the scales have only four items which aim to reflect construct via adjectives while also aiming to avoid the conceptual redundancy/the use of tautological items. In order to be consistent with other works, we proceed with using the observed summed scores in further analyses, but we emphasize that some of the results should be interpreted with caution.

#### Need Satisfaction

The 50-item Need Satisfaction Inventory (Lester, 1990) was used. The Need Satisfaction Inventory assesses the degree of satisfaction of the five basic needs (Physiological needs, Safety and Security needs, Belonging needs, Esteem needs, and Self-actualization needs) on a six-point scale ranging from 1 = strongly disagree to 6 = strongly agree (note: in the original work, the scale ranged from −3 to +3). Summed scores were used for each need scale. The internal consistency statistics for the effective sample were as follows: α = 0.56/ω_t_ = 0.58 (Physiological needs), α = 0.77/ω_t_ = 0.77 (Safety and Security needs), α = 0.73/ω_t_ = 0.74 (Belonging needs), α = 0.81/ ω_t_ = 0.82 (Esteem), and α = 0.80/ω_t_ = 0.82 (Self-actualization needs). The CFA showed that the five-factor solution for Need Satisfaction Inventory had an acceptable fit, χ2_(1,117,N=399)_ = 3,726.837, *p* < 0.001, CFI = 0.945, TLI = 0.942, RMSEA = 0.077 (90% CI: 0.074–0.079).

It could be observed that some of the scales of the Need Satisfaction Inventory exhibit problematic internal consistency values. However, in order to be in line with past (as well as future) research using these summed scores, we use the summed scales in further analyses; however, we do encourage the reader to be careful in interpreting some of the results.

### Analysis

The analyses were conducted in R software v 4.0.3 (R Core Team, 2021). Internal consistency statistics (Cronbach's alphas and McDonald's omega total statistics) were computed with the *psych* package v 2.1.3 (Revelle, 2021). We also checked the model fit of inventories used to assess the constructs of interest. For that, we ran a series of confirmatory factor analyses with the *lavaan* package v 0.6-8 (Rosseel, 2012). PIU and PSU were modeled as unidimensional, while ANPS-AR and the Need Satisfaction Inventory were modeled as having six and five latent variables, respectively. Of note, this modeling was done only for checking the fit of the theoretical models. In other analyses, manifest variables (summed scores) were used. All item-level data were treated as ordinal, probit loadings and polychoric covariance matrices were used. We used the diagonally weighted least squares estimation method. Model fit was assessed by common benchmarks: the comparative fit index (CFI ≥ 0.90), Tucker-Lewis Index (TLI ≥ 0.90), and root mean square error of approximation (RMSEA ≤ 0.10) indicate acceptable fit (MacCallum et al., 1996; Hooper et al., 2008; Kline, 2015).

Spearman bivariate correlation coefficients (*p*-values adjusted with Holm's method) were computed with the *RcmdrMisc* package v 2.7-1 (Fox, 2020).

We first estimated a Gaussian graphical model (GGM; Epskamp et al., 2018b) using the summed scores for PSU and PIU, primary emotional systems, and need satisfaction variables, and age (control variable). Since (younger) age has been shown to be related to be associated with more problematic digital technology use (Horwood et al., 2021), as well as differences in (some of the) primary emotional systems (Montag et al., 2017, 2020) and need satisfaction—life outcomes links (Weman-Josefsson et al., 2015; Wörtler et al., 2020), it would be natural to control the network model for age.

The edges in GGM are conditionally dependent relationships between nodes; in other words, if two nodes are associated, this is after adjusting for associations with all other nodes. The graphical least absolute shrinkage and selection operator in combination with Extended Bayesian Information Criterion (EBICglasso) model selection was used to estimate GGM (Epskamp and Fried, 2018) for a parsimonious, sparse network. The network was estimated with the *bootnet* package v 1.4.3 (Epskamp et al., 2018a). Then, each node was predicted from other nodes for node predictability statistics using the *mgm* package v 1.2-10 (Haslbeck and Waldorp, 2020).

Functionality of *qgraph* v 1.6.9 (Epskamp et al., 2012) was used to plot the network graph. We used the automatically generated layout based on the Fruchterman-Reingold algorithm (Fruchterman and Reingold, 1991). The *qgraph* package c 1.6.9 was also used to plot the strength of nodes. We used the *bootnet* package v 1.4.3 (Epskamp et al., 2018a) for several subsequent network accuracy and stability analyses. In order to assess accuracy of the strength centrality estimates (a), we conducted the routine implemented in the *bootnet* package v 1.4.3 using case-drop bootstrapping based on 1,000 bootstrap samples. We also used the bootstrapped difference-test to ensure interpretable differences in (b) centrality and (c) edge weights. Moreover, (d) we computed the centrality stability coefficient. The network accuracy and stability results of the network are presented in Supplementary Materials, following the reporting guidelines outlined in Burger et al. (2020).

## Results

### Descriptive Statistics and Correlation Analysis Results

Descriptive statistics as well as correlation coefficients (with *p*-values adjusted with Holm's method) for the variables in the focus of this study are presented in Table 1. Of note, while correlations were computed for all pairs of variables, Table 1 only includes the coefficients for correlations that include PIU and PSU, as this is the primary focus of this study. Complete correlation matrix can be found in Supplementary Table 1.

**Table 1:** Descriptive statistics and Spearman correlation analysis results.

**Variable**	***M***	**SD**	**Min**	**Max**	***r* (age)**	***r* (PSU)**	***r* (PIU)**	**cor diff (PSU-PIU) *p***
1. PSU	25.60	12.23	10	60	−0.183^**^	–	0.667^***^	–
2. PIU	28.99	10.38	12	63	−0.205^**^	0.667^***^	–	–
3. SEEKING	20.89	3.65	8	28	0.100	−0.131	−0.203^**^	0.298
4. FEAR	15.81	5.99	4	28	−0.072	0.310^***^	0.309^***^	0.989
5. CARING	20.35	4.13	5	28	0.049	−0.015	−0.094	0.266
6. ANGER	14.30	4.43	4	27	−0.054	0.218^***^	0.168^*^	0.467
7. PLAY	21.35	4.03	7	28	0.006	0.061	−0.052	0.111
8. SADNESS	16.53	5.52	4	28	−0.160^*^	0.174^*^	0.286^***^	0.096
9. Physiological^a^	34.58	6.50	12	54	0.052	−0.296^***^	−0.352^***^	0.381
10. Safety and security	39.70	8.37	14	58	0.064	−0.294^***^	−0.342^***^	0.457
11. Belonging	38.14	8.67	13	56	0.063	−0.013	−0.192^**^	0.011^*^
12. Esteem	40.63	8.08	15	60	0.175^*^	−0.261^***^	−0.389^***^	0.043^*^
13. Self-actualization	39.45	9.00	11	60	0.078	−0.122	−0.296^***^	0.010^*^
14. Age	26.29	7.59	18	55		−0.183^**^	−0.205^**^	0.751

*Min and Max are the empirical minimum and maximum scores. PSU, problematic smartphone use; PIU, problematic Internet use; cor diff p = correlation difference test (using Fisher's r-to-z transformation) between PSU and PIU*.

a *Of note, one item from Physiological needs subscale was excluded from the analyses due to a coding issue (item 16)*.

* *p < 0.05*,

** *p < 0.01*,

*** *p < 0.001; p-values corrected with the Holm's method in r(PSU) and r(PIU)*.

Table 1 shows that both PIU and PSU are correlated with primary emotional systems that reflect the tendency to experience negative affect (FEAR, ANGER, SADNESS). In addition, people with higher scores on the PSU scale have significantly lower scores on physiological, safety and security, and esteem needs. It is necessary to note, however, that all correlation effect sizes were rather small (*r* < 0.300, with the exception of *r* = 0.310 for PSU-FEAR correlation). PIU's relationships with other variables resembled the links observed with PSU. However, in addition, higher PIU was associated with lower SEEKING. Higher scores on the PIU scale were associated with lower scores on all need satisfaction scales. Younger age was associated with higher PSU and PIU scores. Finally, when the difference of correlations was tested (using Fisher's r-to-z transformation), PIU associations, in comparison to PSU's links, were stronger with belonging, esteem, and self-actualization needs satisfaction.

### Network Analysis Results

**Figure 1:**
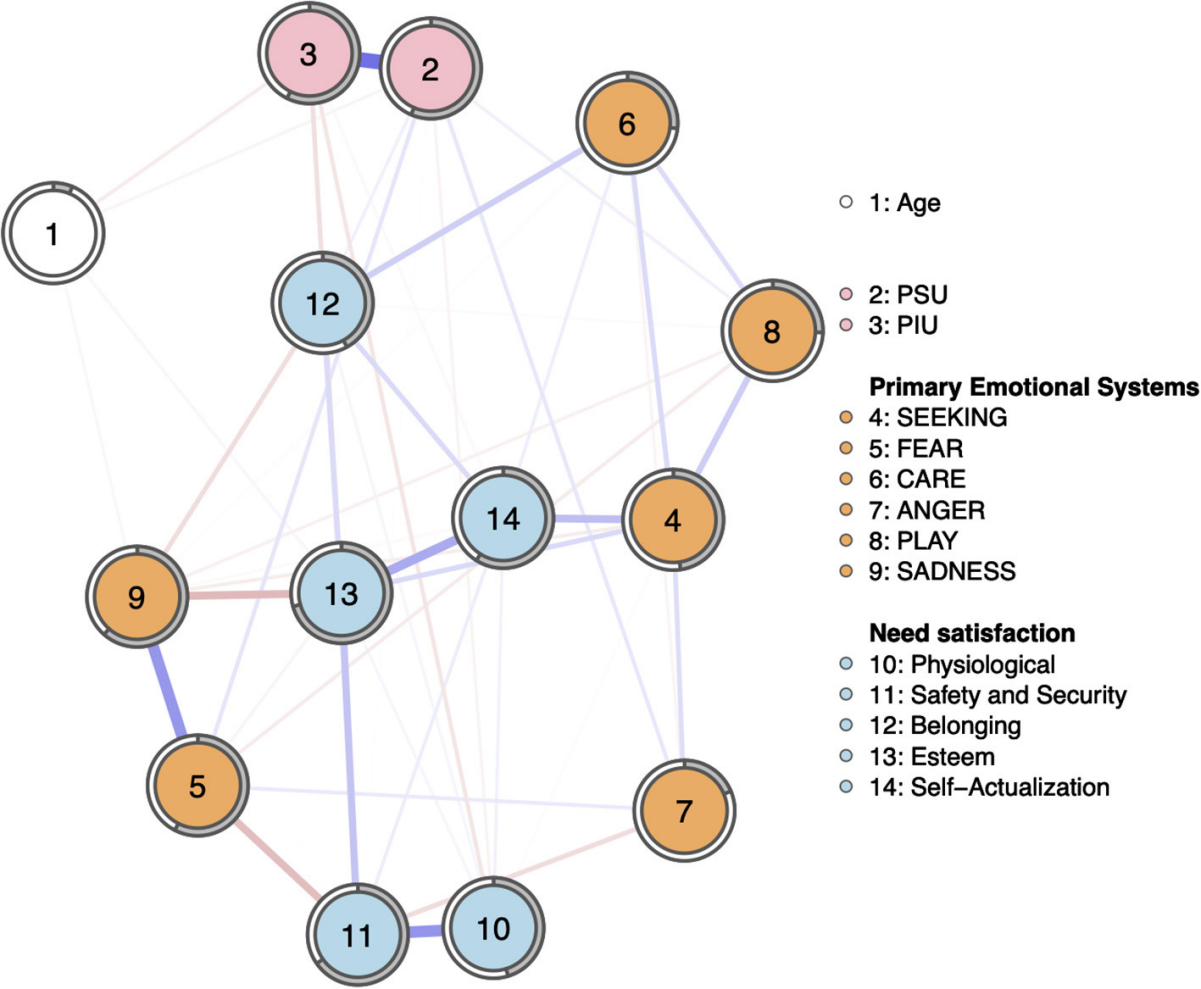
Regularized partial correlation network (tuning-parameter gamma = 0.5). Blue edges represent positive, red edges represent negative regularized partial-correlations. Thickness of line indicates strength of relationship. The values of edges are in Supplementary Table 2. The gray “pie” chart surrounding each node depicts the proportion of a given node's variance explained by other nodes in the network.

The regularized partial correlation network alongside with the predictability of individual nodes is presented in Figure 1. All edge weights depicted in Figure 1 are also provided in Supplementary Table 2.

Network accuracy and stability results are presented in the Supplementary Materials. Overall, the accuracy of edge weights for the estimated model was acceptable (Supplementary Figures 1, 4) and the stability of the network is satisfactory. This is also evidenced by the centrality stability coefficient CS = 0.75 which is large, indicating that the estimated strength was robust (Epskamp et al., 2018a).

The average predictability of nodes in the network was *R*^2^ = 0.458. The predictability for PSU and PIU were *R*^2^ = 0.565 and *R*^2^ = 0.574, respectively. It should be noted, however, that the strong association between PSU and PIU may account for the high predictability of both nodes.

Figure 1 and Supplementary Table 2 shows that PIU and PSU have a strong association with each other. Age is negatively associated with PSU, PIU, and SADNESS, and positively with self-esteem need satisfaction. PSU is positively associated with FEAR, ANGER, PLAY, and belonging need satisfaction, and negatively with physiological and safety needs satisfaction. PIU has negative links with physiological, safety, esteem, and self-actualization needs satisfaction.

Besides the associations with PIU and PSU, it may be interesting to note that several primary emotional systems are correlated with need satisfaction. For instance, SEEKING is positively correlated with self-actualization need satisfaction, while CARE is linked to the satisfaction of belonging needs. On the other hand, FEAR is negatively related to safety and security, and more SADNESS to lower satisfaction of esteem and belonging needs.

**Figure 2:**
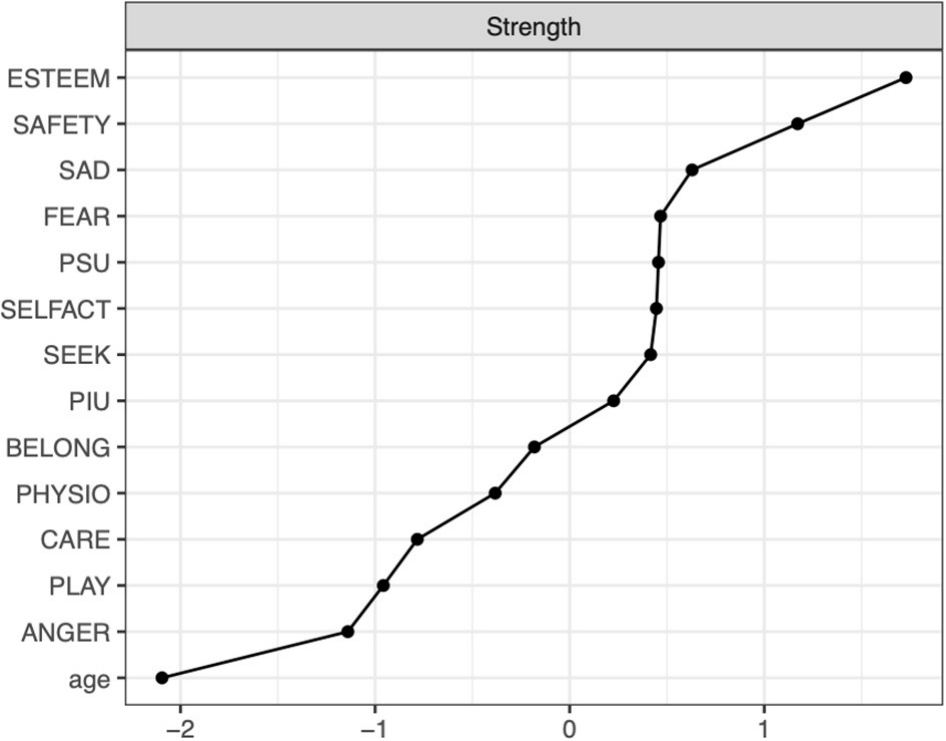
Centrality plots denoting standardized strength results.

Centrality indices for all nodes in the network are in Figure 2. Figure 2 shows that the highest values for strength were for the esteem need satisfaction (but it's not statistically significantly larger than the node strength of safety and security, see Supplementary Figure 3), while the lowest values were for age, ANGER, PLAY, and CARE primary emotional systems. PSU and PIU yield a node strength of roughly equal magnitude—this is also evidenced by these nodes not having a statistically significantly different node strength (Supplementary Figure 3). These results may imply that esteem and safety and security needs satisfaction are central in this network model.

Both PSU and PIU had a negative association with safety and security, and physiological needs satisfaction; however, these associations were not statistically significantly different from each other (Supplementary Figure 2).

## Discussion

The aim of the current work was to investigate the associations between problematic Internet and smartphone use, primary emotional systems, and need satisfaction. In order to meet this goal, we analyzed these variables in bivariate as well as multivariate, network analytic models. Although previous findings provide some evidence for hypothesis testing, our work was largely exploratory.

Bivariate correlation analysis showed that, in general, PIU and PSU are similarly associated with the primary emotional system as well as need satisfaction variables. The associations with negative affect related primary emotional systems (FEAR, ANGER, SADNESS) is not surprising—there is an abundance of evidence linking PIU and PSU with relevant psychopathology, transdiagnostic, and personality variables. For instance, the association between higher depression and anxiety severity and PIU and PSU is robustly demonstrated (Moreno et al., 2015; Elhai et al., 2017; Li et al., 2017). Higher PIU was also associated with lower SEEKING. One potential explanation could be that people who experience higher PIU may also be more depressed (as also demonstrated by literature). Lower SEEKING together with higher SADNESS/FEAR has been linked to more depressive tendencies (Montag et al., 2017). Therefore, there could be an interplay between these three variables—PIU, depression, and SEEKING—that may warrant further attention. These results would also be in line with the I-PACE model (Brand et al., 2019), as individual differences in traits may interact with unsatisfied needs which could, in turn, drive problematic digital technology use. Problematic digital technology use may, in turn, further influence one's need satisfaction, but could also affect one's traits. Even though traits by definition should be relatively stable over time (and, hence, not easily changed), there is evidence that digital interventions could change personality traits (Stieger et al., 2021). It may be that problematic exposure to digital technology use, therefore, could affect personality traits, too.

Another interesting finding in bivariate analyses was that PIU had a stronger negative association with several needs satisfaction than PSU. PSU has been considered one facet of PIU (Baggio et al., 2018); in addition, it could be argued that PSU is a mobile form of PIU (Montag et al., 2021b; see also comments by Elhai et al., 2021). Therefore, it may be that PIU as a wider phenomenon affects—or is affected by—more unsatisfied needs.

When PIU and PSU, primary emotional systems, and need satisfaction variables were modeled in a network model, the results confirmed the relatively strong association between PIU and PSU; where both of these variables were correlated with the same variable (e.g., safety and security as well as physiological needs), these associations did not significantly differ from each other.

Although the effect sizes for associations were small, PIU was conditionally independent of primary emotional systems, while it was negatively associated with some of the need satisfaction variables, namely, physiological, esteem, safety and security, and self-actualization need satisfaction. As mentioned earlier, PIU may be a phenomenon that could affect daily life more—since PSU is bound to a specific medium by definition, PIU could be nurtured via other mediums, too (e.g., PC, tablet, etc.). Furthermore, recent studies have linked PSU primarily to messenger-type application usage (Sha et al., 2019; Rozgonjuk et al., 2020b)—while these applications can also be used as a desktop version, it is likely that communication applications are the main functionality of smartphones. On the other hand, devices with bigger screens may engage their users more, since it is, for instance, more pleasant to watch videos and movies on a big screen. Similarly, screen resolution (and monitor size) may be more relevant in gaming.

On the other hand, PSU was positively linked to primary emotion systems regarding negative affect (FEAR and ANGER) and PLAY. While the findings regarding FEAR and ANGER are also discussed above, it is interesting that the positive association between PSU and PLAY emerged in the network. Of course, it should be noted that the link is weak. Nevertheless, it could mean that smartphones may stimulate PLAY via social contact and smartphone notifications which may function as cues for social contact. People who score higher on PLAY could be more engaged in more problematic smartphone use, as they may expect more (frequent) social cues. These cues are prompted largely in an unexpected frequency (and number), aligning well with the most effective schedule of reinforcement of behavior as seen in Skinnerian schedules of reinforcement (Ferster and Skinner, 1957; Eyal, 2014).

Another interesting finding from the network analysis regarding PSU's links with other variables is that higher PSU is associated with lower physiological needs satisfaction and higher belonging need satisfaction. The association between PSU and belonging need satisfaction may be more straightforward and could align with findings previously discussed with PLAY. It could be that since smartphones are, in essence, tools for communication that have programmable features, PSU may also reflect that this tool is utilized for communication. On the other hand, the findings regarding PSU's and PIU's negative association with physiological needs satisfaction could be due to potentially poorer sleep quality that has been found to be associated with problematic digital technology use (Chen and Gau, 2016; Amez et al., 2020; Wong et al., 2020).

All in all, PSU and PIU were not particularly important nodes in the network—more important were esteem and safety and security needs satisfaction. It may be that when these needs are satisfied, the structure of the network changes. This could also mean that perhaps when these needs are targeted, one's own PSU and PIU levels decrease. Yet, this hypothesis needs to be tested in subsequent works.

Importantly, the primary emotional systems and need satisfaction variables explained a significant proportion of PSU's and PIU's variance—although it seems that most of the technology use variables' variance was explained by the strong association that PSU and PIU had. Even though one may consider these variables equivalent, they seem not to be; this is evidenced by the results of the current study as well as previous empirical findings (Baggio et al., 2018).

The main contribution of this study is providing an overview of PIU's and PSU's associations with primary emotional systems and needs satisfaction. This is the first time that these variables were investigated in a joint network analytic model. The actionable contribution is that perhaps targeting esteem and safety and security needs could result in lower problematic digital technology use scores (see also robust cross-cultural evidence on low self-esteem and higher PIU in Sariyska et al., 2014). In addition, we modeled the associations between these variables as undirected (with regards to causality)—leaving space for scholarly interpretation of the results in terms of causality. The findings of our study also show that modeling choices (e.g., bivariate vs. multivariate) may bring upon different results, showing that bivariate correlations—although arguably informative—may be somewhat misleading. A network analytic approach may provide more valid results.

The main limitations include cross-sectional study design and convenience sampling. While, as mentioned above, the focus of this study was not necessarily on causality, it may nevertheless be of interest to understand the exact dynamics of the associations in the network. While our work does not help out with deterministic cause-and-effect questions, the results provide strong rationale for testing causal hypotheses in future studies. Our sample included people from different (self-reported) countries, and, therefore, cultural differences may play a role in the associations between the key variables in the focus of the study. However, whether this is the case is outside of the scope of the current study—but cross-cultural validation of the results with larger groups per a given country would be useful in resolving this research problem.

## Data Availability Statement

The data and analysis script are available upon scholarly request.

## Ethics Statement

The studies involving human participants were reviewed and approved by Ulm University IRB. Written informed consent for participation was not required for this study in accordance with the national legislation and the institutional requirements.

## Author Contributions

DR and CM: conceptualization. CM: data collection. DR: data analysis and writing (first draft). CM and KD: writing (review and editing). All authors contributed to the article and approved the submitted version.

## Conflict of Interest

KD is employed by Pegasus International Inc. The remaining authors declare that the research was conducted in the absence of any commercial or financial relationships that could be construed as a potential conflict of interest.

## Publisher's Note

All claims expressed in this article are solely those of the authors and do not necessarily represent those of their affiliated organizations, or those of the publisher, the editors and the reviewers. Any product that may be evaluated in this article, or claim that may be made by its manufacturer, is not guaranteed or endorsed by the publisher.
