# Supplementary material for: The Roles of Primary Emotional Systems and Need Satisfaction in Problematic Internet and Smartphone Use: A Network Perspective
Source: Front Psychol. 2021 Aug 31;12:709805. doi: 10.3389/fpsyg.2021.709805 (PMC8438112; doi:10.3389/fpsyg.2021.709805)
Supplement: Supplementary file 1 [file Data_Sheet_1.PDF]

Supplementary Materials

Supplementary Table S1

Bivariate Spearman correlation analysis results

**Variable**													
**PSU**	**PSU**												
**PIU**	.667***	**PIU**											
**SEEK**	-.131	-.203**	**SEEK**										
**FEAR**	.310***	.309***	-.270***	**FEAR**									
**CARE**	-.015	-.094	.349***	-.116	**CARE**								
**ANGER**	.218***	.168*	.104	.247***	-.111	**ANGER**							
**PLAY**	.061	-.052	.357***	-.259***	.282***	.017	**PLAY**						
**SAD**	.174*	.286***	-.413***	.659***	-.273***	.088	-.281***	**SAD**					
**PHYSIO**	-.296***	-.352***	.283***	-.381***	.145	-.180**	.102	-.353***	**PHYSIO**				
**SAFETY**	-.294***	-.342***	.273***	-.580***	.236***	-.279***	.209**	-.507***	.643***	**SAFETY**			
**BELONG**	-.013	-.192**	.332***	-.264***	.365***	-.023	.225***	-.450***	.321***	.403***	**BELONG**		
**ESTEEM**	-.261***	-.389***	.509***	-.539***	.295***	-.095	.201**	-.642***	.475***	.631***	.508***	**ESTEEM**	
**SELFACT**	-.122	-.296***	.542***	-.338***	.325***	-.059	.159*	-.483***	.403***	.498***	.472***	.674***	**SELFACT**
**AGE**	-.183**	-.205**	.100	-.072	.049	-.053	.006	-.160*	.052	.064	.063	.175*	.078

Notes. PSU = problematic smartphone use; PIU = problematic Internet use[1] Of note, one item from Physiological needs subscale was excluded from the analyses due to a coding issue. * p < .05, ** p < .01, *** p < .001. P-value adjusted with Holm's method.

Supplementary Table S2

Edge weights for the network model in Figure 1.

**Variable**													
**AGE**	**AGE**												
**PSU**	-.040	**PSU**											
**PIU**	-.069	.555	**PIU**										
**SEEK**	0	0	0	**SEEK**									
**FEAR**	0	.096	0	0	**FEAR**								
**CARE**	0	0	0	.138	0	**CARE**							
**ANGER**	0	.085	0	.126	.079	-.040	**ANGER**						
**PLAY**	0	.057	0	.187	-.072	.128	0	**PLAY**					
**SAD**	-.031	0	0	-.068	.405	-.014	0	-.054	**SAD**				
**PHYSIO**	0	-.041	-.093	.010	0	0	0	0	0	**PHYSIO**			
**SAFETY**	0	-.005	-.006	0	-.246	0	-.128	0	-.004	.417	**SAFETY**		
**BELONG**	0	.055	0	0	0	.186	0	.014	-.126	.036	.050	**BELONG**	
**ESTEEM**	.032	0	-.118	.140	-.038	0	0	0	-.263	.034	.228	.139	**ESTEEM**
**SELFACT**	0	0	-.023	.253	0	.059	0	0	-.020	.053	.064	.139	.321

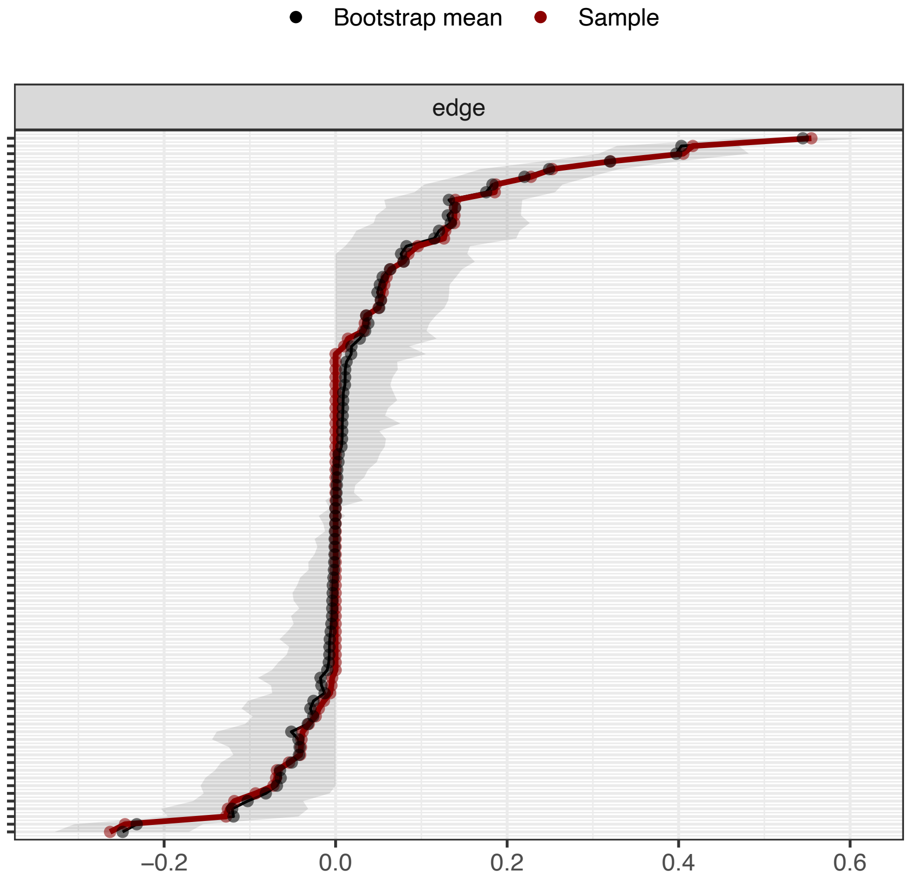

Supplementary Figure S1**.** *Accuracy of the edge-weights for the estimated network depicted in Figure 1.* The horizonal area within the plot represents the 95% quantile range of the parameter values across 1000 bootstraps. The red dots indicate the sample values, while the black dots indicate the bootstrap mean values.

As can be observed in Supplementary Figure S1, the accuracy of the edge weights for the estimated model was acceptable, as indicated by the alignment of the dots for sample and bootstrap mean values. This said, it could also be seen that for some edge weights, the confidence intervals were larger, posing restrictions on interpretation of those edge weight sizes. Therefore, the order of edge estimates should therefore be interpreted with caution.

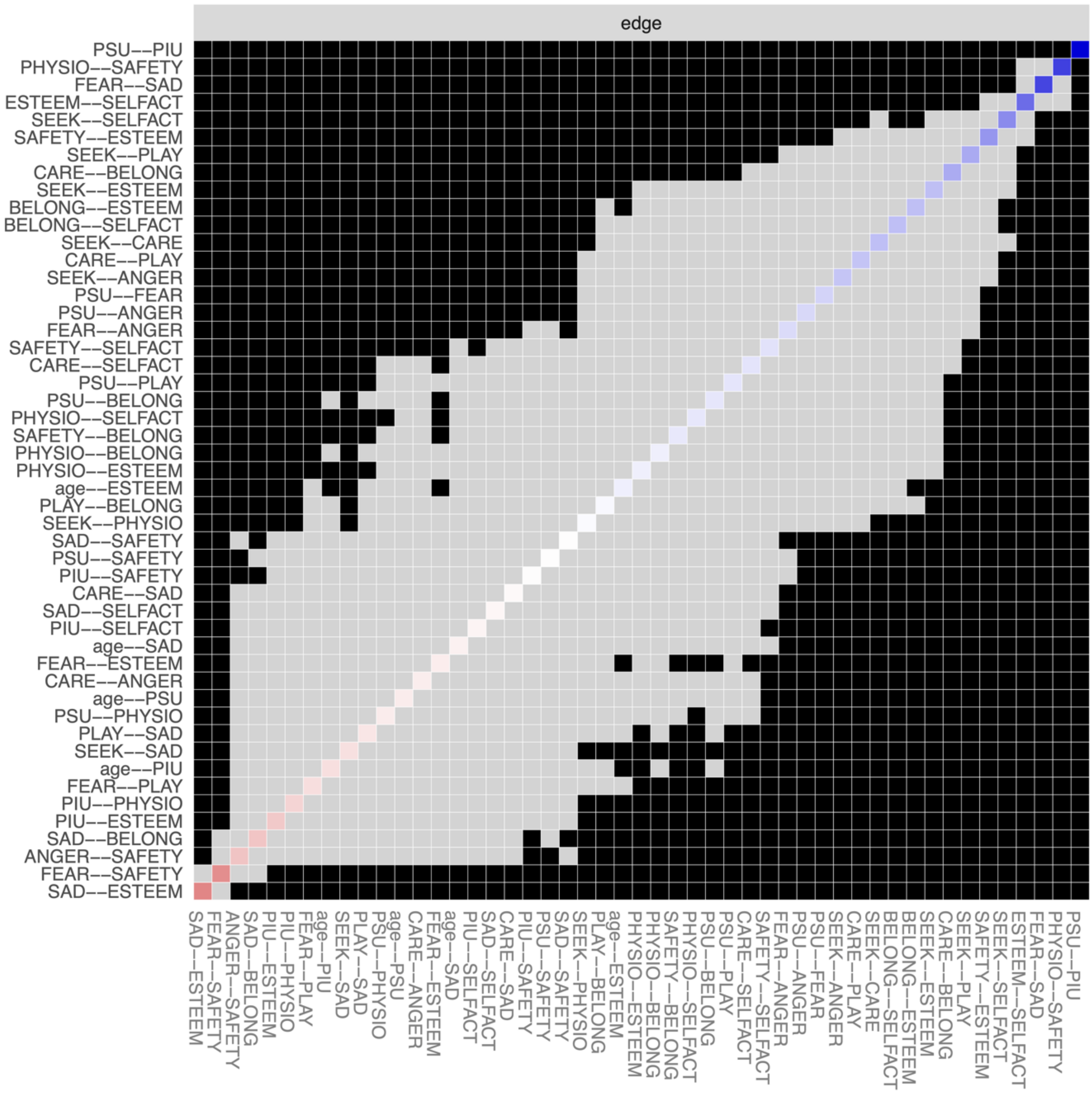

Supplementary Figure S2. *Edge bootstrapped difference test for all non-zero edges in the network structure (α = 0.05) for network depicted in Figure 1.* Light gray boxes reflect no significant differences and dark boxes reflect significant differences. Colored boxes on the diagonal indicate the direction (red = negative; blue = positive) and strength (the more solid the color, the stronger) of the edge in the network depicted in Figure 1.

Supplementary Figure S2 shows that the PSU-PIU edge is significantly different from other edges in the network. However, it would be also interesting to see if PIU and PSU form significantly different associations with other variables in the network. Both PSU and PIU

had an association with safety and security, and physiological needs satisfaction; however, as shown in Supplementary Figure S2, these associations were not statistically significantly different from each other.

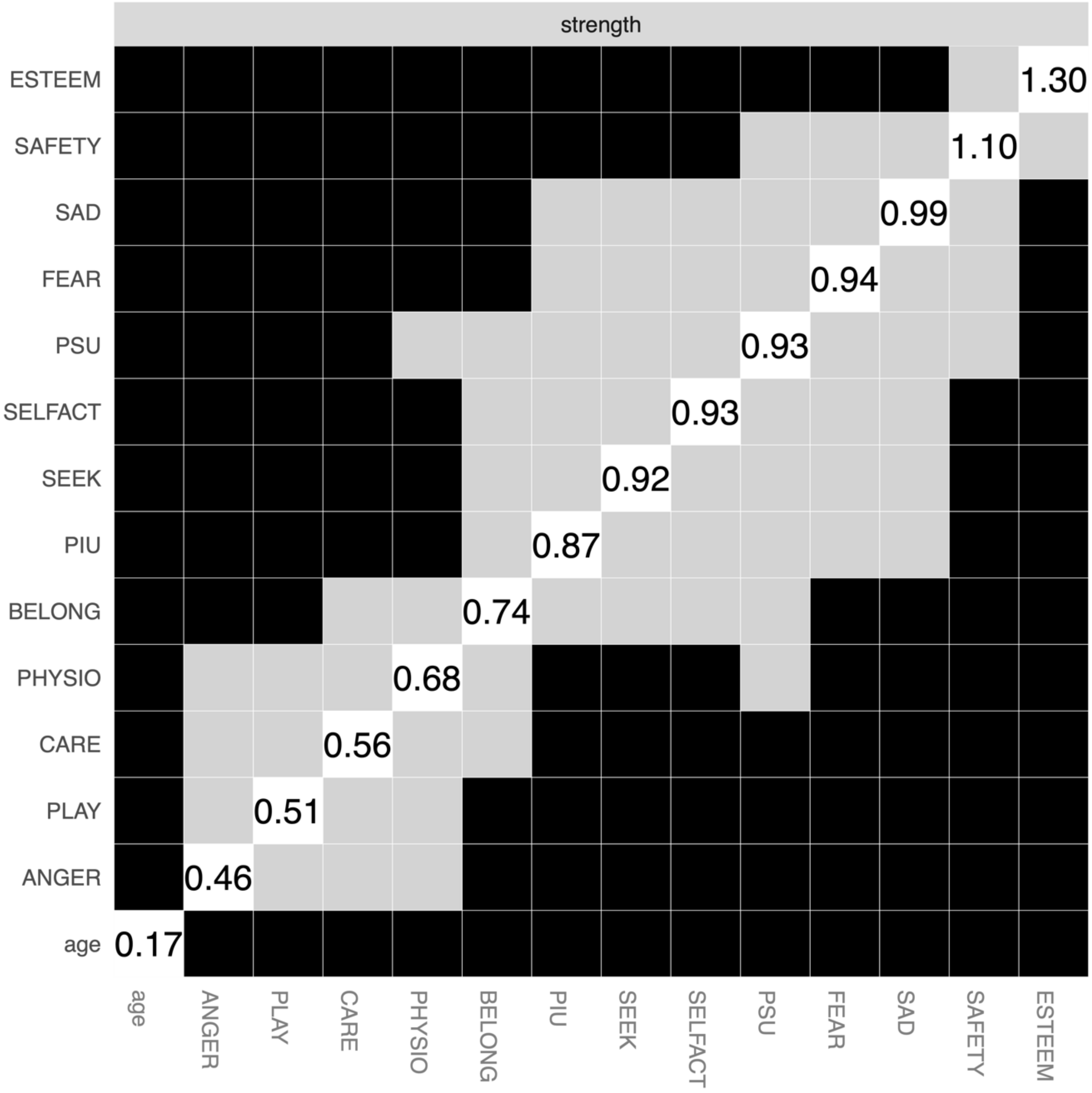

Supplementary Figure S3. *Strength bootstrapped difference test (α = 0.05) for network depicted in Figure 1.* Grey boxes reflect no significant differences and black boxes reflect significant differences.

As could be observed from Supplementary Figure S3, Esteem need satisfaction yields the highest node strength; however, it is not statistically different from Safety and Security need satisfaction node strength. The lowest node strength is for age. PSU and PIU yield a node strength of roughly equal magnitude – this is also evidenced by these nodes not having a statistically significantly different node strength.

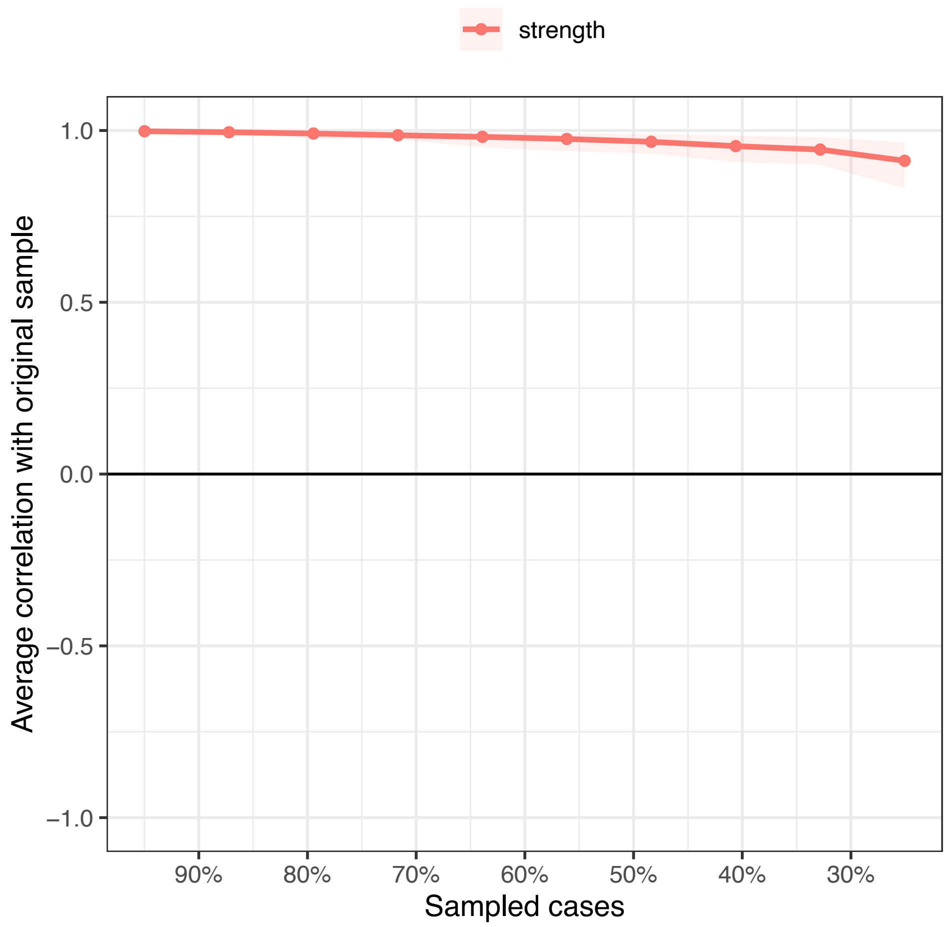

Supplementary Figure S4. *Accuracy of strength centrality estimates for network depicted in Figure 1.*

Overall, the stability of the network is satisfying. This is evidenced by the centrality stability coefficient CS = .75, which is large, indicating that the estimated strength was robust. In other words, the CS indicates that 75% of the data could be dropped to retain with 95% certainty a correlation of $r = .70$ with the original dataset. This is also evidence in Supplementary Figure S4 where different proportions of sample are dropped, but the average correlation with the original sample remains still very high.
